# CRISPR/ddCas12a-based programmable and accurate gene regulation

**DOI:** 10.1038/s41421-019-0085-y

**Published:** 2019-03-12

**Authors:** Jingman Wang, Anrui Lu, Jinxin Bei, Guoping Zhao, Jin Wang

**Affiliations:** 1 The State Key Laboratory of Oncology in South China, Sun Yat-Sen University Cancer Center, 510060 Guangzhou, China; 2 Carson International Cancer Center, Shenzhen Second People’s Hospital, The First Affiliated Hospital of Shenzhen University, Shenzhen University School of Medicine, 518039 Shenzhen, China; 3 Key Laboratory of Synthetic Biology, Institute of Plant Physiology and Ecology, Shanghai Institutes for Biological Sciences, Chinese Academy of Sciences, 200032 Shanghai, China; 4 Shanghai-MOST Key Laboratory of Health and Disease Genomics, Chinese National Human Genome Center at Shanghai, 200000 Shanghai, China; 5 College of Life and Environment Sciences, Shanghai Normal University, 200234 Shanghai, China

**Keywords:** Transcriptional regulatory elements, Gene expression profiling

Dear Editor,

The ability to regulate gene expression may facilitate the study of gene functions underlying complex biological processes and has great potential in both basic biological research and clinical applications^1^. Although customized DNA-binding proteins such as zinc-finger proteins and transcription activator-like effectors (TALEs) have been used as tools for sequence specific DNA targeting and gene regulation, the complexity of the design limits their applications^2^. Recently, due to its much convenience and high efficiency, the CRISPR/Cas9 system has been widely applicated in both genome editing and programmable regulation of gene transcription in many organisms^3,4^. Besides of Cas9, the class 2 type V-A CRISPR-Cas12a offers additional capabilities with better performances including shorter CRISPR RNAs (crRNAs) and the RNase activity for pre-crRNA maturation, facilitating multiplex gene editing^5,6^. Moreover, the DNase-dead Cas12a (namely ddCas12a) can also function as a platform for programming diverse types of transcriptional regulation or epigenetic manipulation of the genome, without changing the genome sequence^7^. However, to accurately control the transcription of a target gene with the CRISPR systems, one may need to either test different targeting sites or choose an inducible CRISPR system^8^, bringing much inconvenience. To solve these problems, we here employed the CRISPR/ddCas12a system that was combined with crRNAs harboring mutated direct repeats (DR) to conveniently regulate gene transcription in a programmable and accurate way.

Based on the crystal structures of the complex of Cas12a and crRNA, the crRNA DR sequences is recognized by Cas12a in a base-specific manner^9^, indicating the importance of the crRNA DR sequences in Cas12a recognition and binding. To further demonstrate this relationship between the DR sequences and the ddCas12a-based transcriptional regulation, we first constructed a reporter system using the *gfp* gene as the reporter. We designed two crRNAs targeting the promoter region and four crRNAs targeting the coding region of *gfp* (Fig. 1a). Both the repression of the *gfp* transcriptional level as determined by quantitative RT-PCR (qRT-PCR) and the reduction of the *gfp* fluorescence signal proved the effectiveness of the above crRNAs. However, crRNAs targeting to the promoter region and to the template DNA strand were much more effective in gene silencing (Fig. 1b), which was consistent with the previous studies^7^. Because the *gfp* fluorescence signal had a good correlation with the qRT-PCR results (e.g., with an *R*^2^ value of 0.9953) (Fig. 1c), the fluorescence signal can be employed to accurately reflect the transcriptional level of *gfp,* as well as the repressive efficiency of the CRISPR/ddCas12a system.

**Fig. 1:**
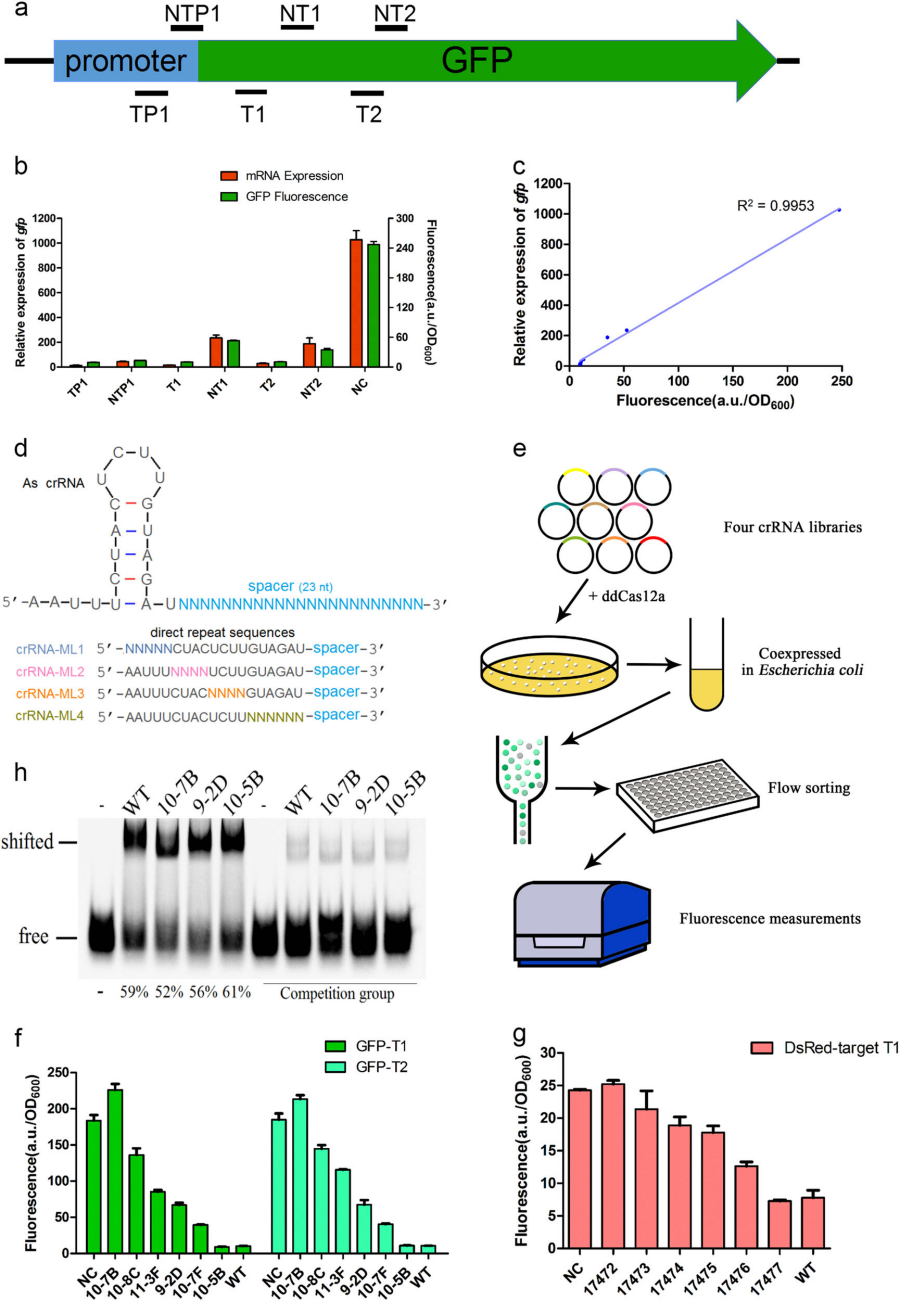
Accurate regulation of gene transcription by the CRISPR/ddCas12a system. **a** Illustration of the positions of crRNAs designed for repression of *gfp* transcription. **b** ddCas12a-mediated repression of *gfp* transcription in *E. coli* MG1655. Both the *gfp* mRNA transcriptional level and the fluorescence intensities were measured, both of which demonstrated the effectiveness of repression by crRNAs targeting to the promoter region or the T strand of *gfp*. Cells expressing ddCas12a and the empty vector pTC17401 were employed as a control, where the transcriptional level of *gfp* was normalized to 1000 and the fluorescence intensities were normalized using the OD_600_ value. **c** Correlation analysis between the *gfp* transcription and the *gfp* fluorescence intensities detected in Fig. 1b. Both data were analyzed using the mean value, with an *R*^2^ value of 0.9953. **d** Illustration of the four mutant libraries with random mutations within the 19-nt crRNA DR sequences. The wild type crRNA DR structure for ddCas12a was shown above, and four mutant libraries (i.e., crRNA-ML1 to crRNA-ML4) were shown below, where “N” in the mutant libraries represented randomized nucleotide substitution on the corresponding position. **e** Illustrative chart of the library screening using the flow sorting method. The obtained mutant libraries were co-transformed with ddCas12a in *E. coli*, followed by the flow sorting according to the fluorescence signal intensities. **f** Quantitative repression of *gfp* transcription with the screened mutant crRNAs. Totally six mutant crRNAs (i.e., 10–7B, 10–8 C, 11–3 F, 9–2D, 10–7 F and 10–5B) were used for analysis, and two distinct targeting sites (i.e., T1 and T2) on *gfp* were tested, employing cells expressing ddCas12a and the empty vector pTC17401 as a negative control or ddCas12a and the wild type crRNA as a positive control. The fluorescence intensities were measured by Varioskan Flash and normalized with the OD_600_ value. **g** Quantitative repression of *DsRed* transcription. Mutant crRNA DR sequences were the same as those in Fig. 1f, and the trends of the repression efficiencies by each mutant crRNA were the similar to those of *gfp* repression. **h** Analysis of the binding affinities of ddCas12a against mutated crRNAs and target dsDNA. Three mutant crRNAs (i.e., 10–5B, 9–2D and 10–7B), which targeted to the T1 site of *gfp* and showed different efficiencies for ddCas12a-based repression, were used for EMSA analysis. The ratio of shifted crRNA to total labeled crRNA probes was calculated by the fluorescence intensity and shown below each lane, and the binding affinities were the highest for 10–5B and lowest for 10–7B, which were consistent with those of the repression efficiencies. The reaction using the wild type crRNA, ddCas12a and target dsDNA was considered as a positive control, while the wild type crRNA itself (i.e., without the addition of ddCas12a and target dsDNA) was used as a negative control and marked as ‘−’. In the competition group, 50 folds of unlabeled crRNA was added into each reaction assay

We then constructed libraries with randomized crRNA DR sequences and used the crRNA-T1 targeting site to analyze the repressive efficiency of each mutated crRNA. Considering the extremely large capacity of the library with totally mutated DR sequences, we divided the crRNA DR sequences into four parts and constructed four corresponding libraries (Fig. 1d). We then co-expressed ddCas12a and each mutated crRNA in *Escherichia coli* and sorted the cells by the fluorescence signal intensity with a flow cytometer (Fig. 1e). In all four mutated libraries, the signal intensity conformed to an approximate normal distribution (Supplementary Fig. S1), but the library of ML3 with mutations in the crRNA DR loop region had the strongest efficiency of transcriptional repression and showed a wider dynamic range of gene transcriptional regulation, indicating mutants in the crRNA DR loop region were more suitable for the development of ddCas12a-based accurate transcriptional regulation systems. Sorted clones were then individually cultured for fluorescence measurement, and clones with different fluorescence intensities were chosen for further analysis of the DR sequences by Sanger sequencing. The secondary structure of the mutated crRNA was predicted using the Mfold program^10^, and crRNAs with strong repressive efficiencies (i.e., low fluorescence intensity) showed a similar secondary structure of DR with that of the wild type (Supplementary Fig. S2).

To test the accuracy and repeatability of the sorted mutated crRNAs, several crRNAs representing a large range of repressive efficiencies were then co-expressed with ddCas12a in *E. coli*, with similar repressive efficiencies obtained on the basis of the *gfp* fluorescence intensities (Supplementary Fig. S3). In addition, we also used the crRNA-T2 targeting site in *gfp* to analyze the generality of the relationship between the crRNA DR sequences and the repressive efficiencies by CRISPR/ddCas12a, and found the same DR sequence showed similar trends in repressive efficiencies between crRNA-T2 and crRNA-T1 (Fig. 1f). Moreover, mutated crRNAs were also designed to target the *DsRed* gene, and the repressive efficiencies were both determined by the *DsRed* fluorescence intensities and judged by the cell color. As expected, similar trends in repressive efficiencies were obtained between *DsRed* and *gfp* with each tested mutant crRNA (Fig. 1g; Supplementary Fig. S4). However, we also found that some mutated clones (e.g., 10–7B) even showed slightly higher fluorescence intensities than that with the empty vector pTC17401, which indicated that the CRIPSR/ddCas system acted as a weak activator instead of a repressor in these clones. It is possible that ddCas12a forms weak complexes with these mutant crRNAs and double-stranded target DNA (dsDNA), which fails to act as a road block but in turn may help the melting of the dsDNA nearby the binding sites, facilitating target gene transcription and therefore functioning as an activator. Besides, although the regulatory efficiency of the CRISPR/ddCas system can be affected by the guide sequences, the same trend of efficiency was found with the mutant crRNAs regardless of different target genes. Based on this finding, we used mutant crRNAs to regulate the transcriptional level of an endogenous gene of *proP* in *E. coli*, and *proP* transcription was regulated by mutant crRNAs to different extents as expected (Supplementary Fig. S5). Besides, as an internal control, transcription of another endogenous gene of *rpoE*, which was not targeted by the crRNAs, was also analyzed and found to be almost unchanged in all tested cells.

To determine the mechanism of the corresponding relationship between the crRNA DR sequences and the repressive efficiencies of ddCas12a, we then used the electrophoretic mobility shift assay (EMSA) to analyze the binding affinities of ddCas12a against mutated crRNAs and target dsDNA, employing either labeled crRNAs (Fig. 1h) or labeled target dsDNA (Supplementary Fig. S6). As expected, the ddCas12a binding affinities against crRNAs and target dsDNA were consistent with their repressive efficiencies, where ddCas12a showed higher affinities with crRNAs that had higher repressive efficiencies (e.g., 10–5B), but lower affinities with less effective crRNA mutants (e.g., 10–7B).

Taken together, we demonstrate here that mutations in the crRNA DR sequences could allow both controllable and quantitative regulation of gene transcription, which may become useful for metabolic engineering of cell factories. For example, the metabolic burden can be minimized by controllable regulation of the corresponding pathways to maximize the yield of target products^11^. Furthermore, this accurate strategy in gene regulation can be combined with the RNase activity of ddCas12a, which allows the generation of multiple mature crRNAs from a crRNA array, facilitating quantitative regulation of multiple genes^7,8^.

## Supplementary information

Supplementary Information

## Supplementary information

**Supplementary Information** accompanies the paper at (10.1038/s41421-019-0085-y).
