# Supplementary material for: CRISPR/ddCas12a-based programmable and accurate gene regulation
Source: Cell Discov. 2019 Mar 12;5:15. doi: 10.1038/s41421-019-0085-y (PMC6411887; doi:10.1038/s41421-019-0085-y)
Supplement: Supplementary file 1 — Supplementary Information [file 41421_2019_85_MOESM1_ESM.pdf]

Supplementary information

Materials and Methods

Plasmid construction

The ddCas12a expression plasmid with *gfp* reporter gene (namely pTC17058-GFP) was constructed, containing a strong constitutive promoter (J23100) for ddCas12a expression, a strong constitutive promoter (J23101) for *gfp* expression, a chloramphenicol selection marker and a p15A replication origin. With the Ezmax seamless cloning kit (Tolo Biotech., Shanghai, China), ddCas12a gene, *gfp* gene and plasmid backbones were seamlessly assembled and verified by sequencing. For the *DsRed* reporter plasmid, the *DsRed* gene was cloned into pTC17058-GFP to replace the *gfp* gene, obtaining pTC17058-DsRed. For ddCas12a protein expression and purification, the E993A mutation was introduced by PCR amplification using a pair of primers on the basis of pET28a-TEV-AsCas12a[1], followed by *Dpn*I treatment and direct transformation into DH10B competent cells. The whole ddCas12a gene was verified by Sanger sequencing before being used for protein expression and purification.

The crRNA transcription vector was constructed on the basis of pgRNA-bacteria[2], namely pTC17401. Notably, two *Bbs*I sites were introduced after the J23119-SpeI promoter, which facilitate further insertion of different guide sequences. To insert one guide sequence, a pair of oligos were heat denatured and annealed, before being inserted into the *Bbs*I-linearized pTC17401. All inserted crRNA guide sequences were verified by Sanger sequencing with primer pcrRNA-ZF.

All primers used in this study were listed in Supplementary Table S1.

Real-time reverse transcription (RT)-PCR

Strains were grown overnight in liquid LB medium with appropriate antibiotics at 37 °C with shaking at 220 rpm, then 50 μl culture was inoculated into 5 ml fresh liquid LB medium and further incubated till the OD_{600} reached 1.2 for cell harvesting. Total RNA was extracted using the ZR Fungal/Bacterial RNA MiniPrep (Zymo Research, Irvine, CA, USA), and further treated with RNase-free DNase I (TaKaRa, Shiga, Japan) to prevent the contamination of trace genomic DNA. RT was performed using the

PrimeScript II 1st Strand cDNA Synthesis Kit (TaKaRa), and PCR was carried out with the reaction mixture (from 50 ng RNA) as the template, using the SYBR qPCR master mix (TaKaRa) and the StepOne Plus Real-Time PCR machine (Thermo Fisher Scientific). The *gapA* gene was employed as an internal control for analysis of samples. Three independent samples were employed for analyses.

Fluorescence assay

Strains were grown in liquid LB medium with appropriate antibiotics at 37 °C overnight with shaking. Then 100 µl culture was added in 96-well polystyrene plates to subject to fluorescence and OD_{600} measurements using a Varioskan Flash from Thermo Fisher Scientific. *gfp* fluorescence emission was excited at 488 nm and detected at 507 nm, and *DsRed* fluorescence emission was excited at 563 nm and detected at 582 nm. In all the cases, fluorescence was normalized using $OD_{600,}$ and the strains expressing ddCas12a and the empty vector pTC17401 as a negative control or ddCas12a and the wild type crRNA were employed as a positive control. Three biological replicates were used for the fluorescence assays.

Statistical Analysis

All the experiments were performed in triplicate, and the data were analyzed with the GraphPad Prism software (version 5.01, San Diego, CA, USA), with the values represented as mean ± standard error of the mean (SEM).

Flow Cytometry and Analysis

Firstly, we constructed four randomized crRNA libraries and the libraries were constructed by PCR and endonuclease-mediated restriction and ligation. Specifically, the upstream primers contained the randomized nucleotides and the downstream primer was a universal primer (Supplementary Table S1). After PCR amplification, DNA fragments were treated with *Bbs*I and then inserted into the *Bbs*I-treated pTC17401. The ligated products were transformed into MG1655 competent cells harboring the ddCas12a and *gfp* expression plasmid. After transformation, cells were plated on solid LB agar supplemented with antibiotics of ampicillin and chloramphenicol. After ~16 h of growth, >10^5 cells were collected and pooled, diluted into fresh liquid LB medium with antibiotics, and cultured for 1 h. Cultures were then diluted into liquid LB medium

to sort the cells by fluorescence signal intensity on a MoFlo XDP Flow Cytometer (Beckman Coulter, USA). Sorted cells were cultured at 37 °C overnight with shaking and then subject to fluorescence and OD_{600} measurements.

RNA secondary structure analysis

The RNA secondary structural models of the crRNA DR sequences were constructed by the Mfold program, and the energy minimization program of Mfold website server (http://mfold.rna.albany.edu/?q=mfold/RNA-Folding-Form) under default folding conditions (37 °C, 1 M NaCl, no divalent ions and no limit on distance between paired bases) was used[3].

Electrophoretic mobility shift assays

Internally labelled crRNA probes were first prepared by *in vitro* transcription. Briefly, the transcription templates were prepared through annealing of the synthesized oligonucleotides with T7-crRNA-F (Supplementary Table S1) as previously described[1,4]. And 500 ng template DNA was used to transcribe the corresponding crRNA in a 50 µl reaction mix with or without 1 nmol Cy5-UTP using a T7 High Yield Transcription Kit (Thermo Fisher Scientific), and the reaction was performed at 37 °C overnight (approximately 16 h). Then, both unlabeled and Cy5-labelled crRNAs were purified using the RNA Clean & Concentrator™-5 (Zymo Research) and quantified with the NanoDrop 2000C (Thermo Fisher Scientific). The ddCas12a protein was expressed and purified as described previously[1].

For binding reactions using labelled crRNAs, 1.5 µg ddCas12a protein and 1 µg target dsDNA of 302 bps in length were incubated with 100 ng Cy5-crRNA probe at 37 °C for 30 min in NEB Cutsmart buffer in a final volume of 20 µl. For the competition assay, 5 µg unlabeled crRNA (50 ×) was added to the above reaction system containing 100 ng Cy5-crRNA probe. Then, 10-µl reaction mix was loaded onto a 5% non-denaturing polyacrylamide gel and electrophoresis was performed by running at 150 V for 20 min in 1× TAE buffer (40 mM Tris-Acetic acid, 2 mM EDTA [pH 8.0]). Gels were scanned by a FLA-9000 phosphoimager (FujiFilm Corporation, Japan).

For binding reactions using labelled target dsDNA, two complementary

oligonucleotides were synthesized, annealed in 1 × Taq DNA polymerase buffer and then extended by Taq DNA polymerase, and the obtained product was used as the template for subsequent PCR amplification using 6-Carboxyfluorescein (6-FAM)-labelled primers of M13F-47 and M13R-48 (*ref to* Supplementary Table S1). Then, 2 μg ddCas12a protein and 200 ng crRNAs were incubated with 150 ng FAM-labelled target dsDNA probe at 37 °C for 30 min in NEB Cutsmart buffer in a final volume of 20 μl. For competition group, 7.5 μg unlabeled target dsDNA (50 ×) was added to the above reaction system containing 150 ng FAM-labelled target dsDNA. After incubation, 10-μl reaction mix was loaded onto a 1.5% agarose gel and electrophoresis was performed by running at 150 V for 12 min in 1× TAE buffer (40 mM Tris-Acetic acid, 2 mM EDTA [pH 8.0]). Gels were scanned by the ImageQuant LAS 4000 mini system (GE Healthcare, USA).

Supplementary Table

Supplementary Table S1. Oligonucleotides for plasmid construction and crRNA plasmid preparation

Oligo names	Sequences (5'-3')
p15A-CMF	aggatccccaggcatcaaataaaacg
p15A-CMR	ctaggactgagctagccgtctatgtgtgggagggctaacc
ddCas12a-F	cacacatagacggctagctcagtcctaggtacagtg
ddCas12a-R	aatgtagcacctgaagtcagc
GFP-F	gacttcaggtgctacatttttacagctagctcagtcctag
GFP-R	ttgatgcctggggatccttatttgtatagttcatccatgccatg
DsRed-F	aaagaggagaaggatccatggataacaccgaagatgtg
DsRed-R	atgcctggggatccttactggctgccgctatg
DsRed-VectorF	agtaaggatccccaggcatc
DsRed-VectorR	ggatccttctcctctttaatctc
17401F	tttgtcttcgagagaagaccttttttttgaagcttgggcccgaac
17401R	aggtcttctctcgaagacaaactagtattatacctaggactgagc
pcrRNA-ZF	gtatcaacagggacaccag
AsCpf1-E993A-F	tcaggttggccagcaccaccacggcctggtag
AsCpf1-E993A-R	ggtgctggccaacctgaatttcggctttaagag
GFPcrRNA-TP1F	tagtaatttctactcttgtagatcagctagctcagtcctaggtatt
GFPcrRNA-TP1R	aaaaaatacctaggactgagctagctgatctacaagagtagaaatt
GFPcrRNA-NTP1F	tagtaatttctactcttgtagatatctctagagctagcataatacc
GFPcrRNA-NTP1R	aaaaggtattatgctagctctagagatatctacaagagtagaaatt
GFPcrRNA-T1F	tagtaatttctactcttgtagatactggagttgtcccaattcttgt
GFPcrRNA-T1R	aaaaacaagaattgggacaactccagtatctacaagagtagaaatt
GFPcrRNA-T2F	tagtaatttctactcttgtagataaggtgatacccttgttaataga
GFPcrRNA-T2R	aaaatctattaacaagggtatcaccttatctacaagagtagaaatt
GFPcrRNA-NT1F	tagtaatttctactcttgtagattgcccattaacatcaccatctaa
GFPcrRNA-NT1R	aaaattagatggtgatgttaatgggcaatctacaagagtagaaatt
GFPcrRNA-NT2F	tagtaatttctactcttgtagatactcgattctattaacaagggta
GFPcrRNA-NT2R	aaaatacccttgttaatagaatcgagtatctacaagagtagaaatt
10-7B- GFPcrRNA-T2F	tagtaatttctacgccagtagataaggtgatacccttgttaataga
10-7B- GFPcrRNA-T2R	aaaatctattaacaagggtatcaccttatctactggcgtagaaatt
10-8C- GFPcrRNA-T2F	tagtaatttctacggttgtagataaggtgatacccttgttaataga
10-8C- GFPcrRNA-T2R	aaaatctattaacaagggtatcaccttatctacaaccgtagaaatt
11-3F- GFPcrRNA-T2F	tagtaatttctacgtgtgtagataaggtgatacccttgttaataga
11-3F- GFPcrRNA-T2R	aaaatctattaacaagggtatcaccttatctacacacgtagaaatt
9-2D- GFPcrRNA-T2F	tagtaatttctacctcggtagataaggtgatacccttgttaataga
9-2D- GFPcrRNA-T2R	aaaatctattaacaagggtatcaccttatctaccgaggtagaaatt
10-7F- GFPcrRNA-T2F	tagtaatttctacataggtagataaggtgatacccttgttaataga

10-7F- GFPcrRNA-T2R	aaaatctattaacaagggtatcaccttatctacctatgtagaaatt
10-5B- GFPcrRNA-T2F	tagtaatttctactttggtagataaggtgatacccttgttaataga
10-5B- GFPcrRNA-T2R	aaaatctattaacaagggtatcaccttatctaccaaagtagaaatt
10-7B- DsRedcrRNA-T1F	tagtaatttctacgccagtagataagtgcgcatggaaggcagcgtg
10-7B- DsRedcrRNA-T1R	aaaacacgctgccttccatgcgcacttatctactggcgtagaaatt
10-8C- DsRedcrRNA-T1F	tagtaatttctacggttgtagataagtgcgcatggaaggcagcgtg
10-8C- DsRedcrRNA-T1R	aaaacacgctgccttccatgcgcacttatctacaaccgtagaaatt
11-3F- DsRedcrRNA-T1F	tagtaatttctacgtgtgtagataagtgcgcatggaaggcagcgtg
11-3F- DsRedcrRNA-T1R	aaaacacgctgccttccatgcgcacttatctacacacgtagaaatt
9-2D- DsRedcrRNA-T1F	tagtaatttctacctcggtagataagtgcgcatggaaggcagcgtg
9-2D- DsRedcrRNA-T1R	aaaacacgctgccttccatgcgcacttatctaccgaggtagaaatt
10-7F- DsRedcrRNA-T1F	tagtaatttctacataggtagataagtgcgcatggaaggcagcgtg
10-7F- DsRedcrRNA-T1R	aaaacacgctgccttccatgcgcacttatctacctatgtagaaatt
10-5B- DsRedcrRNA-T1F	tagtaatttctactttggtagataagtgcgcatggaaggcagcgtg
10-5B- DsRedcrRNA-T1R	aaaacacgctgccttccatgcgcacttatctaccaaagtagaaatt
DsRedcrRNA-T1F	tagtaatttctactcttgtagataagtgcgcatggaaggcagcgtg
DsRedcrRNA-T1R	aaaacacgctgccttccatgcgcacttatctacaagagtagaaatt
10-7B- proP-T1F	tagtaatttctacgccagtagatttgcttacgcattaggtaaagtt
10-7B- proP -T1R	aaaaaactttacctaatgcgtaagcaaatctactggcgtagaaatt
11-3F- proP -T1F	tagtaatttctacgtgtgtagatttgcttacgcattaggtaaagtt
11-3F- proP -T1R	aaaaaactttacctaatgcgtaagcaaatctacacacgtagaaatt
9-2D- proP -T1F	tagtaatttctacctcggtagatttgcttacgcattaggtaaagtt
9-2D- proP -T1R	aaaaaactttacctaatgcgtaagcaaatctaccgaggtagaaatt
10-5B- proP -T1F	tagtaatttctactttggtagatttgcttacgcattaggtaaagtt
10-5B- proP -T1R	aaaaaactttacctaatgcgtaagcaaatctaccaaagtagaaatt
GFP-Target DNA-302F	caggagctgaggaactagtac
GFP-Target DNA-302R	ccgaaagtagtgacaagtgttg
Labelled target dsDNA-F	cgccagggttttcccagtcacgacagaacttttcactggagttgtccc aattcttg
Labelled target dsDNA-R	gcggataacaatttcacacaggacatctaattcaacaagaattggga caactccag
FAM-M13F(-47)	FAM-cgccagggttttcccagtcacgac
FAM-M13R(-48)	FAM-agcggataacaatttcacacagga
T7-crRNA-F	gaaattaatacgactcactataggg
EMSA-10-7B-GFPcrRNA-T1F	acaagaattgggacaactccagtatctactggcgtagaaattcccta tagtgagtcgtattaatttc
EMSA-9-2D-GFPcrRNA-T1R	acaagaattgggacaactccagtatctaccgaggtagaaattcccta tagtgagtcgtattaatttc
EMSA-10-5B-GFPcrRNA-T1R	acaagaattgggacaactccagtatctaccaaagtagaaattcccta tagtgagtcgtattaatttc
EMSA- GFPcrRNA-T1R	acaagaattgggacaactccagtatctacaagagtagaaattcccta tagtgagtcgtattaatttc

crRNA-ML1F	ctgcgagagaagaccttagtNNNNNctactcttgtagatactggagttgtcccaattct
crRNA-ML2F	ctgcgagagaagaccttagtaatttNNNNtcttgtagatactggagttgtcccaattct
crRNA-ML3F	ctgcgagagaagaccttagtaatttctacNNNNgtagatactggagttgtcccaattct
crRNA-ML4F	ctgcgagagaagaccttagtaatttctactcttNNNNNNactggagttgtcccaattct
crRNA-MLR	ctggcggcgaagaccgaaaaacaagaattgggacaactccagt
GFP-qPCR-F	ctactttcggttatggtgttc
GFP-qPCR-R	gtcttgtagttcccgtcatc
proP-qPCR-F	cgatgttcccgacgcatatc
proP-qPCR-F	catcaggtaataggcaggcatc
rpoE-qPCR-F	acatggctgtatcggattgc
rpoE-qPCR-F	aatggcatccacatcactgg

Supplementary Figures

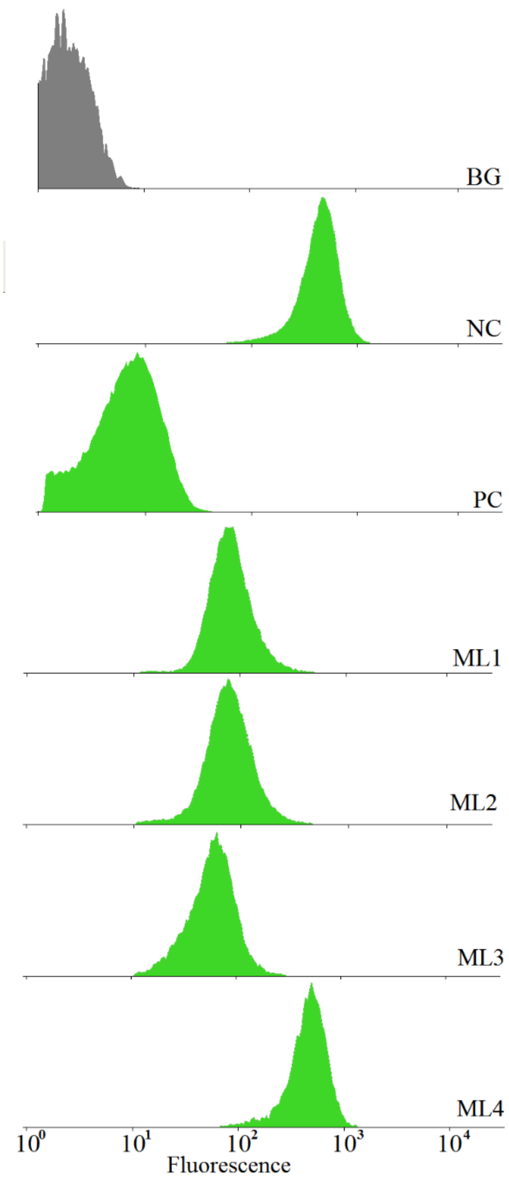

Supplementary Fig. S1. Sorting of mutant crRNA libraries using flow cytometry. Repression of the *gfp* gene in MG1655 was performed by ddCas12a and a mutant crRNA, targeting the T1 site in *gfp*, and cells were sorted on the basis of the fluorescence value. BG: *E. coli* MG1655 strain serving as the background; NC: the reporter strain harboring the empty vector (pTC17401); PC: the reporter strain harboring the wild type crRNA targeting the T1 site in *gfp*. For four crRNAs libraries, ML3 had the strongest efficiency of transcriptional repression, ML1 and ML2 had the middle efficiency, whereas ML4 had the lowest efficiency.

a

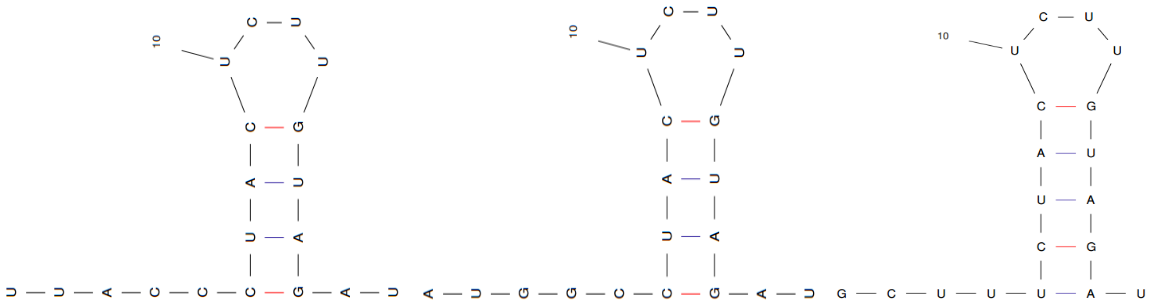

1-8G
Fluorescence (a.u./OD_{600})=239.1212

4-12F
Fluorescence (a.u./OD_{600})=94.70406

5-6E
Fluorescence (a.u./OD_{600})=48.54802

b

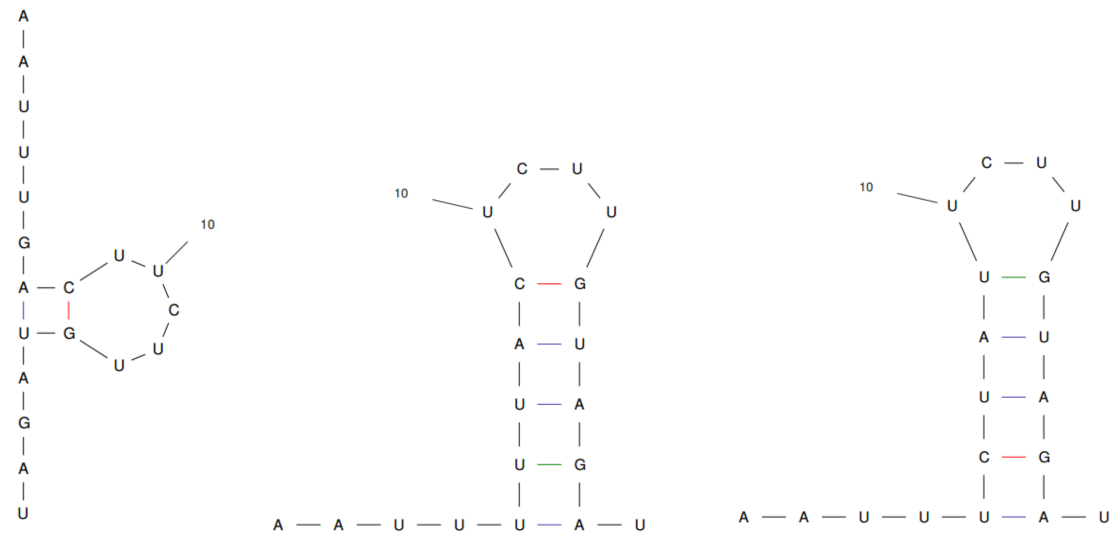

2-11G
Fluorescence (a.u./OD_{600})=206.2806

7-10B
Fluorescence (a.u./OD_{600})=90.68263

8-1H
Fluorescence (a.u./OD_{600})=32.78977

c

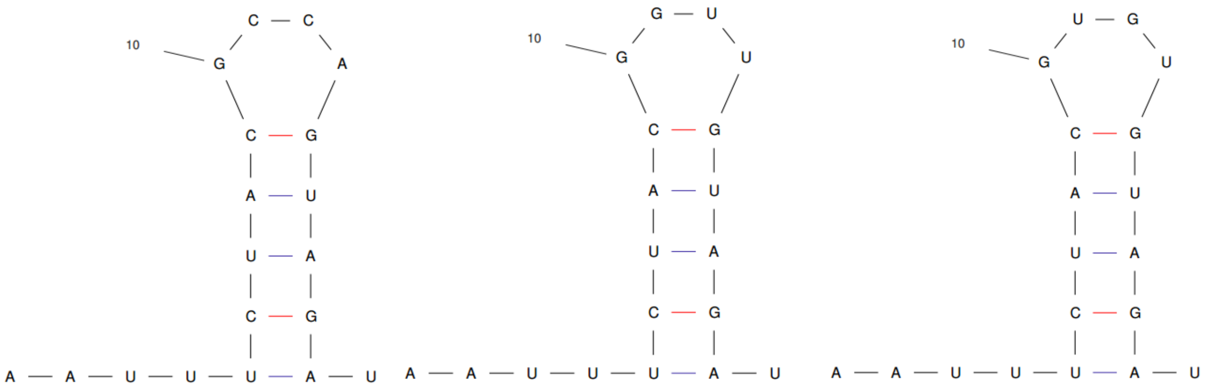

10-7B
Fluorescence (a.u./OD_{600})=225.95

10-8C
Fluorescence (a.u./OD_{600})=136.086

11-3F
Fluorescence (a.u./OD_{600})=85.2869

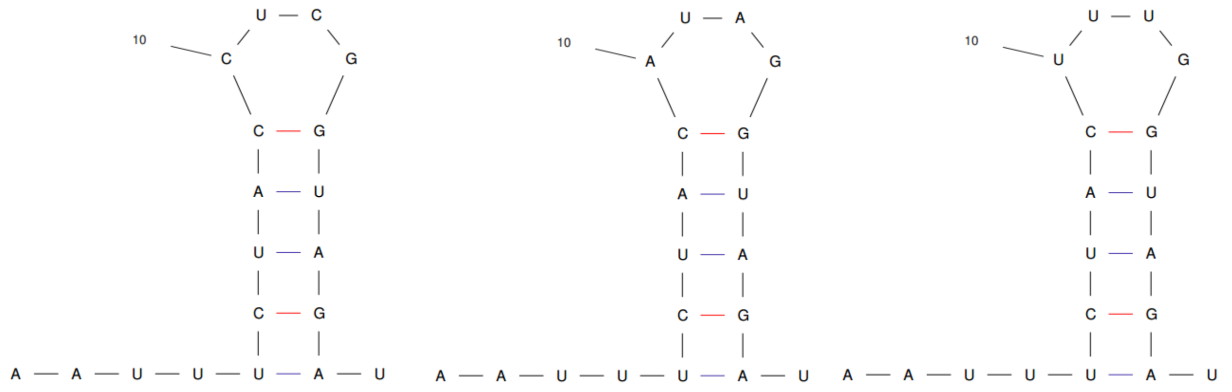

9-2D
Fluorescence (a.u./OD_{600})=66.9574

10-7F
Fluorescence (a.u./OD_{600})=38.9055

10-5B
Fluorescence (a.u./OD_{600})=8.81867

d

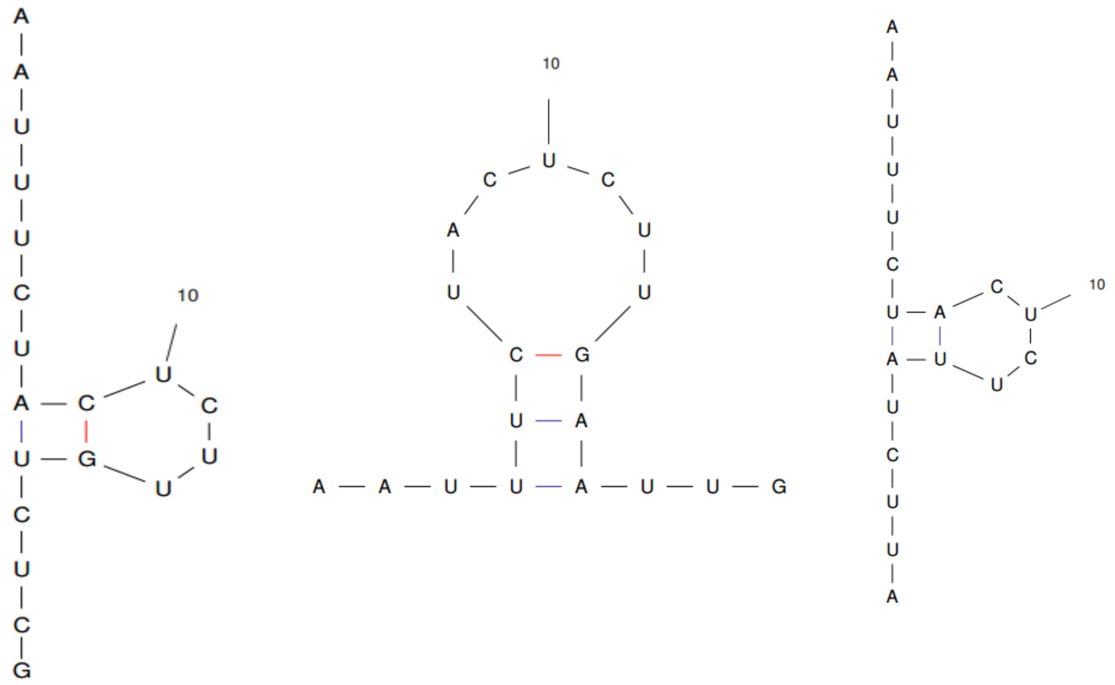

12-8G
Fluorescence (a.u./OD_{600})=250.1208

12-3F
Fluorescence (a.u./OD_{600})=135.2255

14-11G
Fluorescence (a.u./OD_{600})=68.00977

Supplementary Fig. S2. Predicted secondary structures of the crRNA DR sequences screened from the randomized crRNAs libraries by flow sorting. The folding pattern was generated by the Mfold program as described in *Materials and Methods*. The names of the crRNA DR sequences were showed under the structure of the crRNA DR sequences, which was based on the positions of corresponding strains in 96-well polystyrene plates. The crRNAs with the different DR sequences could guide different repression efficiencies by targeting the T1 site of *gfp*, and the values of

fluorescence normalized with OD_{600} were also showed. (a) crRNA DR sequences screened from ML1 crRNAs library. (b) crRNA DR sequences screened from ML2 crRNAs library. (c) crRNA DR sequences screened from ML3 crRNAs library. (d) crRNA DR sequences screened from ML4 crRNAs library.

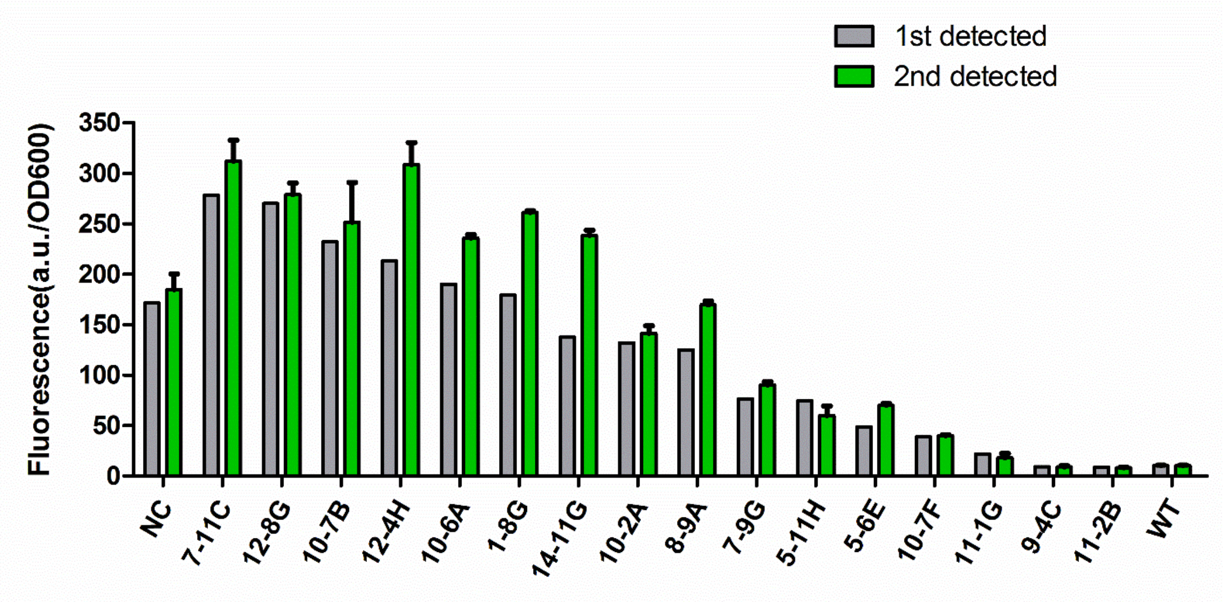

Supplementary Fig. S3. Repressive efficiencies of mutated DR sequences. The first detection was performed after the flow sorting, and the second detection was performed with new *E. coli* MG1655 cells transformed with ddCas12a and extracted crRNA plasmids. The fluorescence signal was normalized using OD_{600}, and the transcription of *gfp* in cells expressing ddCas12a and the empty vector pTC17401 as a negative control (NC) or ddCas12a with the wild type crRNA as a positive control. In the first detection, cells sorted by a flow cytometer were first cultured in 200-μl liquid LB medium in 96-well plates, which were put into a shaker with 100 rpm. In the second detection, cells were inoculated in 5-mL liquid LB medium in a 25-mL test tube, and cultured in a 37 °C shaker with 220 rpm. To detect the fluorescence, 100-μl culture from either the 96-well plate or the test tube was then transferred to a clean 96-well plate for fluorescence measurement.

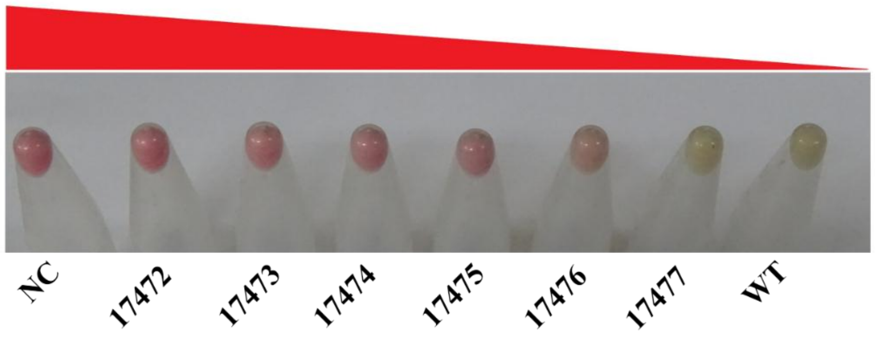

Supplementary Fig. S4. ddCas12a-mediated repression of the transcription of *DsRed* in MG1655. Strains coexpressing the ddCas12a, crRNAs and *DsRed* gene were grown in liquid LB medium with appropriate antibiotics at 37 °C overnight with shaking. Then 3 ml culture was collected to analyze the color changes of bacteria. crRNAs with the different DR sequences can guide different efficiencies of gene transcriptional regulation. The transcription of *DsRed* in cells expressing ddCas12a and the empty vector pTC17401 as a negative control (NC) and ddCas12a with the wild type crRNA as a positive control.

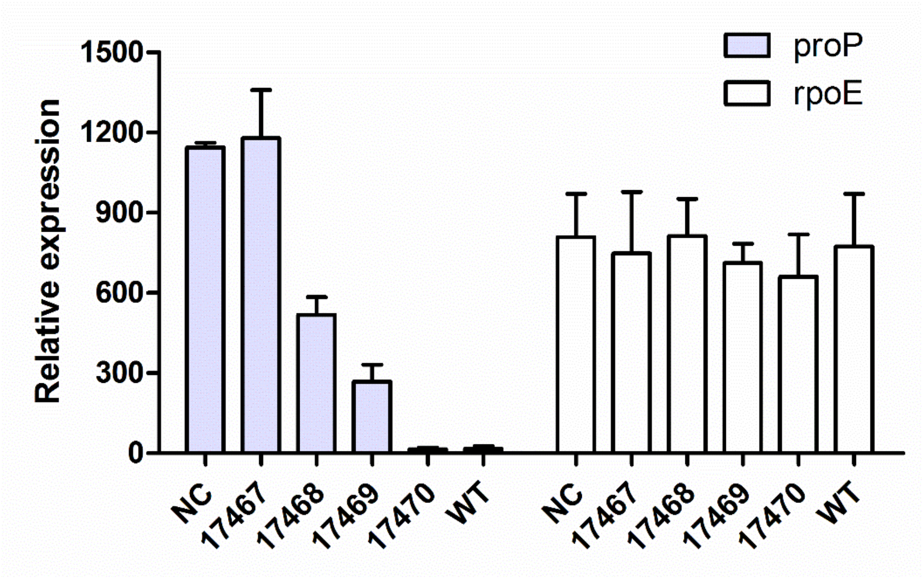

Supplementary Fig. S5. ddCas12a-mediated repression of *proP* transcription in MG1655. crRNAs were designed to target the T strand in the coding region of *proP*, and the transcription of *proP* was determined by real-time RT-PCR. Cells expressing ddCas12a and the empty vector pTC17401 was employed as a negative control (NC), and the transcriptional level of *proP* was normalized to 1000. The *rpoE* gene was not targeted by the tested crRNAs and was therefore employed as an internal control. The

transcriptional level of *rpoE* gene was also individually analyzed in cells expressing distinct crRNAs.

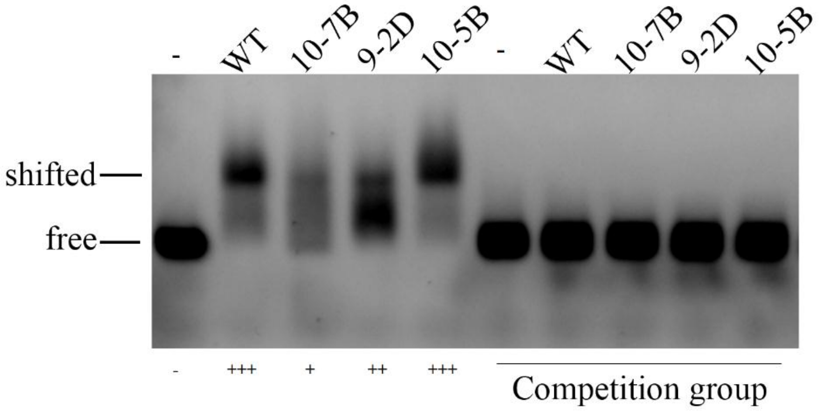

Supplementary Fig. S6. Analysis of the binding affinities of ddCas12a against mutated crRNAs and labelled target dsDNA. Mutant crRNAs used for EMSA analysis here were the same as those in **Fig. 1h**. The reaction employing the wild type crRNA was used as a positive control and was marked as 'WT', while labelled target dsDNA itself (i.e. without the addition of ddCas12a and crRNA) was used as a negative control and was marked as '-'. In the competition group, 50 × unlabelled target dsDNA was first added into each reaction system before the addition of labelled target dsDNA. Consistent with the results in **Fig. 1h** and the repression studies, the binding affinities were the highest for 10-5B and lowest for 10-7B.

References

1 Lei, C. *et al.* The CCTL (Cpf1-assisted Cutting and Taq DNA ligase-assisted Ligation) method for efficient editing of large DNA constructs in vitro. *Nucleic acids research* **45**, e74, doi:10.1093/nar/gkx018 (2017).

2 Qi, L. S. *et al.* Repurposing CRISPR as an RNA-guided platform for sequence-specific control of gene expression. *Cell* **152**, 1173-1183, doi:10.1016/j.cell.2013.02.022 (2013).

3 Zuker, M. Mfold web server for nucleic acid folding and hybridization prediction. *Nucleic acids research* **31**, 3406-3415 (2003).

4 Li, S. Y. *et al.* CRISPR-Cas12a has both cis- and trans-cleavage activities on single-stranded DNA. *Cell research* **28**, 491-493, doi:10.1038/s41422-018-0022-x (2018).
